# Toward Linguistic Recognition of Generalized Anxiety Disorder

**DOI:** 10.3389/fdgth.2022.779039

**Published:** 2022-04-15

**Authors:** Laurens Rook, Maria Chiara Mazza, Iulia Lefter, Frances Brazier

**Affiliations:** Faculty of Technology, Policy and Management, Delft University of Technology, Delft, Netherlands

**Keywords:** generalized anxiety disorder, mental distress, emotion regulation, natural language processing, BIS/BAS

## Abstract

**Background:**

Generalized anxiety disorder (GAD) refers to extreme, uncontrollable, and persistent worry and anxiety. The disorder is known to affect the social functioning and well-being of millions of people, but despite its prevalence and burden to society, it has proven difficult to identify unique behavioral markers. Interestingly, the worrying behavior observed in GAD is argued to stem from a verbal linguistic process. Therefore, the aim of the present study was to investigate if GAD can be predicted from the language people use to put their anxious worries into words. Given the importance of avoidance sensitivity (a higher likelihood to respond anxiously to novel or unexpected triggers) in GAD, this study also explored if prediction accuracy increases when individual differences in behavioral avoidance and approach sensitivity are taken into account.

**Method:**

An expressive writing exercise was used to explore whether GAD can be predicted from linguistic characteristics of written narratives. Specifically, 144 undergraduate student participants were asked to recall an anxious experience during their university life, and describe this experience in written form. Clinically validated behavioral measures for GAD and self-reported sensitivity in behavioral avoidance/inhibition (BIS) and behavioral approach (BAS), were collected. A set of classification experiments was performed to evaluate GAD predictability based on linguistic features, BIS/BAS scores, and a concatenation of the two.

**Results:**

The classification results show that GAD can, indeed, be successfully predicted from anxiety-focused written narratives. Prediction accuracy increased when differences in BIS and BAS were included, which suggests that, under those conditions, negatively valenced emotion words and words relating to social processes could be sufficient for recognition of GAD.

**Conclusions:**

Undergraduate students with a high GAD score can be identified based on their written recollection of an anxious experience during university life. This insight is an important first step toward development of text-based digital health applications and technologies aimed at remote screening for GAD. Future work should investigate the extent to which these results uniquely apply to university campus populations or generalize to other demographics.

## 1. Introduction

Generalized anxiety disorder (GAD) is a common disorder, characterized by constant, unfocused, excessive worrying and anxiety, which increases in intensity with age (1). The International Classification of Diseases (ICD-11) describes GAD as “free-floating anxiety or excessive worry focused on multiple everyday events” (2). In the Diagnostic and Statistical Manual of Mental Disorders (DSM-5), people are diagnosed with GAD, if they report to have experienced three or more of the following symptoms for several days of the week in the past months: restlessness, fatigue, concentration issues, irritability, muscle tension, and/or sleep disturbance (3). In 2011, GAD is assumed to have affected 8.9 million people in the European Union, with major repercussions on social and occupational functioning (4).

In spite of its prevalence and burden to society, the disorder has proven difficult to recognize —the behavioral symptoms and mechanisms underlying GAD are poorly understood (5). A recent mega-analysis of existing data on the brain regions associated with stress responses in people diagnosed with GAD, for instance, did not yield significant differences between the brain structure of people with GAD and controls (6). Nevertheless, researchers, in general, seem to agree on the role that avoidance strategies (i.e., deliberate anxious responses in the face of novel or unexpected stimuli) play in the functioning of people with GAD [cf., (7–10)]. There also appears to exist consensus regarding the importance of anxious worrying behavior in GAD (11). In the avoidance model of worry and GAD—a cornerstone theory in the field—this aspect in GAD is argued to be verbal linguistic in nature. People with GAD appear to be in the habit of putting their worries into words (8). However, the low number of empirical investigations into the verbal linguistic aspects of GAD is a stark contrast to the importance assigned to language in extant theorizing on the disorder. The limited number of studies that have attempted to do so, yielded fragmented and contradictory findings, and did not use the writing prompts for prediction of GAD.

One theoretical explanation for the lack of similarity in findings on language and GAD may be that avoidance strategies, such as those present in worry and GAD, cannot be fully understood in the absence of approach strategies (i.e., deliberate eagerness and anticipation under exposure to novel and unexpected stimuli). In Gray's (12) Reinforcement Sensitivity Theory, the behavioral inhibition system (BIS) is a brain system related to anxiety in response to novel stimuli (avoidance); the behavioral activation/approach system (BAS) was a separate brain system triggered by reward and non-punishment (approach). In later revisions (13), considerable overlap is assumed between the approach and avoidance system. Approach (BAS) and avoidance (BIS) are “joint subsystems” that resolve conflict together, and should not be explored in isolation (14, 15).

Therefore, the research objectives in the present study were 2-fold. First, we intended to explore whether GAD can be predicted from the way in which people put their worry into words. Second, we sought to examine if prediction accuracy in GAD recognition from linguistic responses increases with the inclusion of individual differences in approach and avoidance sensitivity. Gaining insight into these issues would indicate that remote screening for GAD is possible. This would make a contribution to the field of digital health, in the sense that it could initiate a larger research program, focused on systematic investigation of linguistic markers for GAD in a wider range of settings. Such research could eventually lead to the development of digital health applications for at-a-distance identification of GAD in clinical setting.

The remainder of this article is structured as follows: Section 2 reviews the theoretical models of worry and GAD, as well as the extant research into language and GAD. Approach and avoidance sensitivity are discussed from a reinforcement sensitivity theory point of view. Section 3 presents the methodology and data collection. The results are presented in Section 4. The possible contributions of our findings, the limitations, as well as their implications for the remote screening of GAD in digital health applications are addressed in Section 5.

## 2. Theoretical Background

### 2.1. Theoretical Models of Worry and GAD

Researchers have attempted to understand the symptoms, causes, and possible mechanisms underlying GAD from various theoretical angles. In general, the theories can be divided into: (1) cognition-based, (2) emotion-based, and (3) behavioral avoidance models of worry and GAD (16).

First, the anxious worrying behavior characteristic of GAD is often explained cognitively as an intolerance to uncertain and ambiguous threats. In this theoretical approach, people with GAD may lack confidence in their own problem-solving and decision-making capabilities (17, 18). Doubts and worries could especially manifest under exposure to uncertain and ambiguous stimuli that potentially render the person with GAD indecisive and distressed—possibly due to an inherent conflict with the cognitive need for predictability, order and structure that may come with the disorder (19). As a consequence, some people with GAD develop a negative attitude toward problem-solving and decision-making, in general. For some people with GAD, uncertainty about a particular event may trigger maladaptive information processing—a recurrent train of thought characterized by persistent worrying about negative, undesirable, or otherwise problematic consequences of taking action (9, 20). This may eventually lead to cognitive demoralization and exhaustion for review, see Behar et al. (16).

Second, anxious worrying behavior and GAD are posited to stem from maladaptive coping with emotions (10, 21). In this alternative theoretical approach, people with GAD are generally described as being more likely to experience emotional hyperarousal. This could, for instance, manifest in frequent and intense occurrences of negative affect. Research shows that some people with GAD feel uneasy with emotions such as anger, anxiety, sadness. In individual cases, negative beliefs about the general purpose of emotions have been documented (21). The hypervigilance/tension that comes with emotional hyperarousal may invite behaviors aimed at emotion avoidance, specifically (21–23); for a review on this aspect, see (16, 24).

Third, anxious worrying behavior and GAD have been explored from a behavioral avoidance point of view (7, 25). This theoretical approach centers on the notion of excessive worry, which has gained prominence as the defining feature of GAD ever since the proposal by Andrews et al. (11) to rename it Generalized Worry Disorder. In accordance with this, people with GAD are theoretically assumed to display specific behaviors aimed at controlling or preventing excessive worry. Among others, they may engage in acts of cognitive avoidance such as procrastination, rumination, and indecisiveness [as in the intolerance to uncertainty approach of (9)]. Also, they may show safety behaviors, such as careful planning of future actions, and prevention-focused activities undertaken so as not to make any mistakes. Further, people with GAD may display a tendency to engage in acts of reassurance seeking in the opinions of other people (11). Many of the premises of the behavioral avoidance theory of worry and GAD are supported by empirical evidence. Behavioral avoidance strategies do, in general, manifest in people with GAD, and lead them toward excessive worrying behaviors that periodically spin out of control (5, 25, 26).

Despite differences in orientation, a commonality in theorizing (whether grounded in cognition, emotion, or behavioral avoidance) is that people with GAD may find it difficult to deal with , and adequately respond to mental distress triggered by unanticipated stimuli. Their default response is to avoid such stimuli (cognitively, emotionally, or behaviorally), which periodically reinforces worry and GAD.

### 2.2. The Verbal-Linguistic Nature of Worry and GAD

In the avoidance model of worry and GAD (8), worry is considered a verbal linguistic phenomenon, in which unique characteristics of an individual, and the way in which a threat is perceived, determine how worry will be expressed in words (16).

This influential theoretical framework is a hybrid of cognition-based and emotion-centered models, in which GAD is understood as initially provoking worrying behavior in response to an imagined or real threat. People unaffected by GAD, under such circumstances, engage in image-based scrutiny of the potentially alarming threat, but people with GAD, in general, display a tendency to replace the emotional mental imagery with less intrusive verbal-linguistic thoughts (8, 20). While this verbal-linguistic avoidance strategy may seem a wise emotion regulation strategy, it tends to render people with GAD more prone to worry persistently about uncertain or ambiguous emotion-laden events (5, 7, 16, 27).

Empirical research in written form into the verbal linguistic aspects of GAD is sparse. Building on the observation that anxious people in general are more likely to engage in affect-laden, self-focused, processing styles under exposure to acute and chronic distress (28, 29), research among people with GAD shows that self-focus manifests in alleviated use of first-person singular pronouns in written recall of events (30). This is confirmed in research on mental health forums, where the written contributions of people with GAD contain more first-person singular pronouns and less first-person plurals (31). Lyons et al. (31) further show that emotion words, such as positive and negative emotion words, affective process words, anger and—especially—anxiety and sadness, occur frequently in the writings of people with GAD.

Anxiety, sadness and negative emotion words also feature prominently in the titles of the YouTube videos that people with GAD tend to watch online—just as titles that contain social words (friends). The Google Search histories of people with GAD, on the other hand, primarily contain queries on personal concerns (with work, money, and death) (32). Contrasting this, the linkages between first-person singular pronouns, emotion words and GAD were not observed in recent longitudinal work on (spoken) blog content. Instead, a focus on the present emerged as the linguistic feature that most prominently and positively correlates with GAD (33). This finding, however, is at odds with an earlier study in clinical setting, in which the spoken language of women diagnosed with GAD was linked to lower use of present, and higher use of future, verb tense (34). These findings may be attributed to platform-specific characteristics and inherent differences between written and spoken language, but they also illustrate how little consensus exists regarding the verbal linguistic markers of GAD.

One theoretical explanation for the lack of similarity in findings may be that avoidance strategies, such as those present in worry and GAD, cannot be fully understood in the absence of approach strategies. If this is true, the written language of people with GAD may vary further due to additional heterogeneity of individuals in terms of avoidance and approach sensitivity. This possibility is discussed in the next section.

### 2.3. Individual Differences in Self-Regulation

Considerable evidence exists for a link between disorder sensitivity, in general, and individual differences in self-regulation. According to Gray's biopsychological theory of emotion (12), personality is grounded in a behavioral inhibition system (BIS) and a behavioral approach system (BAS). The BIS is a brain system related to anxiety in response to novel stimuli (avoidance); the BAS is a brain system triggered by reward and non-punishment (approach). A third brain system, the fight-flight-freeze system (FFFS), was added in the revised Reinforcement Sensitivity Theory (13), an updated version of Gray's initial theory. The FFFS is activated in immediate fearful response to clear and acute threats [c.f., (15)].

The BIS/BAS scales (35) are validated self-report measures, rooted in Gray's original theory, and developed with the aim to assess individual differences in self-regulation. Research with these self-report scales has established associations with psychiatric disorders. High scorers on BIS are more vulnerable to anxiety disorders, whereas high scorers on BAS are more prone to other disorders. This does not rule out the possibility that a high scorer on BAS is sensitive toward anxiety due to comorbidity (mostly with depression or alcohol dependence) (36). The BIS also plays a role in GAD, as first posited in Johnson et al. (36), and found in a study on anxious vs. non-anxious children, where children high on GAD scored higher on BIS than those low on GAD (37). The finding was replicated in a Japanese student sample (38), and in an adult community sample, in which BIS accurately predicted current GAD status (39).

Reinforcement Sensitivity Theory, the theoretical revision, contains two important changes. The introduction of the FFFS as a third brain system is the most striking modification. The BIS/BAS scales can be used to assess individual FFFS-sensitivities (40), but it does not make sense theoretically to do so for GAD. Fear and anxiety are conceptually distinct phenomena that trigger different behaviors. Fear is a defensive response to a clear threat, whereas anxiety is worrying behavior due to an ambiguous stimulus that *may* be threatening [cf., (14, 41)]. Pharmacological evidence exists, indicating that fear is not related to GAD (42). In line with this, research shows that anxious worrying behavior is counterproductive in physically dangerous tasks that require fast responses under stress—circumstances, characterized by a clear and present threat (13, 43).

The second modification concerns a theoretical reappraisal of the relationship between the approach and avoidance-related system. In the most recent iteration of the theory, the BIS is assigned the role of a gatekeeper system in the detection and resolution of goal-conflict arising from novel or unanticipated stimuli. The BIS critically scans such stimuli for possible issues in a negatively valenced process of worry and rumination, until a verdict is reached. The BIS does not only resolve conflict by applying avoidance considerations; it may also agree to promising, interesting or rewarding (BAS-focused) incentives. The BIS and BAS thus are highly interdependent systems for self-regulation that resolve inner conflict in close interaction with each other (13–15). From a theoretical point of view, it follows that attempts to understand problems and challenges involving avoidance-related forms of self-regulation (such as emotion dysregulation in worry and GAD) should always include approach-related forms of self-regulation.

### 2.4. The Present Study

Motivated by the theoretically recognized verbal linguistic nature of GAD expression and the documented links between GAD and behavioral inhibition, this study focuses on linguistic analysis of written content in relation to GAD. The study seeks to answer, whether GAD can be predicted from linguistic characteristics of expressive writing, and whether GAD prediction accuracy increases, if individual differences in BIS and BAS sensitivity are included. Considerable evidence exists in Computational Science that mental disorders can be identified from physiological, nonverbal and—especially also—verbal signals [cf., (44)]. Such prediction models tend to rely on the ways in which people put the psychological distress they experience into words (45).

GAD has received significantly less attention compared to other mental disorders, and the fragmented work that investigated language and GAD was never undertaken with the explicit aim to automatically recognize this disorder.

Therefore, undergraduate students were asked to write about an anxious experience in their university life. Students are known to more likely suffer from mental health disorders, including anxiety disorders. Moreover, the high cognitive load and formal requirements of the education system, together with the dynamics of campus life, cause university students to experience more mental distress than their non-studying counterparts (46). We reasoned that these particularities would render it likely to find verbal linguistic markers of GAD in the written narratives of undergraduate students. It also made it feasible to assess participants on self-reported GAD, BIS, and BAS sensitivity. This set-up enabled us to collect linguistic and self-report data in a study population that is particularly affected by anxiety-related issues in general, and to perform a set of classification experiments to evaluate GAD predictability based on linguistic features, BIS/BAS scores, and a concatenation of the two.

## 3. Materials and Methods

### 3.1. Data Collection

The research protocol and data management plan for the present study were approved by the Human Research and Ethics Committee of Delft University of Technology, the Netherlands. Informed consent was received from all participants under study. Five expert psychologists from the clinical center on campus were interviewed prior to data collection to secure the appropriate tone and framing for the research project.

### 3.2. Participants

University students were asked to volunteer in online research investigating experiences with university life. The initial sample consisted of 144 participants, but two participants (1.39%) were excluded from the analysis as they did not submit a substantial portion of the text requested. This resulted in a final sample of 142 participants (56 men and 86 women; *M* age = 23.33 years, *SD* = 1.96), that was used for analyses reported in the present study.

### 3.3. Procedure

Students interested in this research project received an URL link powered by Qualtrics™. Upon opening the link, a landing page was shown with a brief introduction on the study, the main instructions, a confidentiality agreement, an informed consent button, and a reminder that at all times the participant had the right to opt out of the study. Having provided informed consent, participants were asked to fill out a short questionnaire comprised of two self-report measures (as introduced in greater detail in the subsection below). Next, participants were invited to work on a writing task. Upon completion, participants received a short post-questionnaire that included demographics. Finally, participants were debriefed and thanked for their participation.

### 3.4. The Writing Task

Participants were invited to work on a narrative writing exercise inspired by the expressive writing paradigm . This writing paradigm was developed to explore individual motives and emotion states in relation to a person's physical and mental health (47, 48). The standard procedure is to ask people to write for some time about personal experiences, events, or topics that describe the need to execute control. In psychotherapy, the writings may be requested in multiple sessions with varying writing instructions—i.e., therapeutic interventions [cf., (49)]. The experience of writing about such episodes in life has shown to exert a small, positive influence on someone's well-being and physiological functioning (50). Inspired by this established writing task, participants in the present study were asked to recollect and vividly describe , in a single writing session, an anxious experience related to their university experience.

In order for us to appropriately frame the writing task, five university psychologists were asked for advice prior to the data collection. They confirmed that undergraduate students on campus do experience problems relating to generalized anxiety. Their recommendation was to start the task with an open inquiry into the student's university experience in general. This technique stems from Cognitive Behavioral Therapy (CBT), and is used by the campus psychologists to form a first impression about the needs of a client at intake. An open-ended question that is unrelated to any critical event in particular allows the client to freely express thoughts and feelings, worries and anxieties. Based on these suggestions, the writing task was introduced as follows:

“*What do you think about your university experience? Moreover, can you describe an anxious moment during your university life, and how this made you feel?”*

One example of an expressive writing response from a person with a high GAD score was:

“*[…] I had several anxious moments during my university experience. Particularly when I had to make some decision, or before an exam, or waiting for the results. Particularly now, when I see that I need to find a job and I do not find it, I feel lost, hopeless, I feel stupid as nothing I did was enough. I am so worried to be nobody and I cannot relate to this. I have difficulties sleeping, in being happy, I am sad! […] I am so worried”*.

One example of an expressive writing response from someone with a low GAD score was:

“*During the mid half of the […] bachelor program […] I started to feel stressed due to multiple projects, thinking about the future, i.e., whether I want to start a job or whether I want to study further and where I should then apply. During the last year […] the pressure of the final year project and thesis work made for many restless and sleepless nights. But what keeps me motivated and focused in the end are my goals and ambitions. Moreover, university or student life experience overall is exciting and fun no matter the difficult or tough times the individual subjects bring up. I learned a lot of things apart from course books that cannot be replaced from anything, and has shaped me better in areas which are proving to be beneficial in my professional life”*.

The minimum word count for the writings was set at 100 words, which was consistent with previous studies (51). The data collection resulted in 142 valid texts with an average length of 165 words, and a median of 140 words.

### 3.5. Measures

Two validated self-report measures were administered in the questionnaire component of the study: (1) the GAD-7, which is a scale that measures sensitivity toward generalized anxiety disorder, and (2) the BIS/BAS scales that tap individual differences in approach-avoidance sensitivity. The characteristics of these two measurement instruments are described in this subsection.

#### 3.5.1. GAD

To measure each student's level of generalized anxiety disorder, the GAD-7 scale was used (52). The scale is based on the diagnostic criteria that were formulated in the DSM-IV (53), and is composed of seven items that together assess the amount of anxiety experienced in the previous weeks, on a 4-point scale anchored at 0 (*not sure at all*) and 3 (*nearly every day*) for each item. Respondents are asked to reply to GAD problems such as: “Feeling nervous, anxious, or on edge” (item 1), and “Worrying too much on different things” (item 3). The reliability coefficients for the overall GAD-7 score in this sample were high (Cronbach's α = 0.90, Guttman's λ6 = 0.90, McDonald's ω = 0.91). The summation of answers on the seven items in the GAD-7 scale results in a minimum score of 0 and a maximum score of 21. It is uncommon to use the resulting sum score as an overall GAD severity measure. As instructed in Spitzer et al. (52) and Kroenke et al. (53) ,the sum score must be converted into a categorical (GAD/no GAD) label, based on a cut-point ≥10. This is a validated split value, unaffected by age, sex, recruitment stratum, and linguistic background (54), which is applied to clinical samples (55) as well as to the general population for review, see Kroenke et al. (56). Participants under study reported an average anxiety score of *M* = 8.95 (*SD* = 5.79) on the GAD-7. Based on the validated cut-point, 55 undergraduate students (38.73%) in our sample fell in the GAD category.

#### 3.5.2. BIS- and BAS-Sensitivity

The BIS/BAS scales were used to measure individual differences in BIS- and BAS-sensitivity (35). These scales consist of 24 items (7 BIS-items, 13 BAS-items, and 4 filler items) measured on a 4-point scale anchored at 1 (*very true for me*) and 4 (*very false for me*). Examples of BIS items are: “Even if something bad is about to happen to me, I rarely experience fear or nervousness” (item 2; reverse-scored), and “I worry about making mistakes” (item 24). The reliability coefficients for the BIS in this sample were satisfactory (Cronbach's α = 0.87, Guttman's λ6 = 0.89, McDonald's ω = 0.87). Examples of BAS items are: “I go out of my way to get things I want” (item 3), “I'm always willing to try something new if I think it will be fun” (item 5). Also the reliability coefficients for the BAS items in this sample were acceptable (Cronbach's α = 0.82, Guttman's λ6 = 0.86, McDonald's ω = 0.83).

### 3.6. Statistical Analyses

#### 3.6.1. LIWC Categories

The texts written by the participants were analyzed using the Language Inventory Word Count (LIWC; version 2015; Text Analysis Portal for Research, University of Alberta) (57). LIWC 2015 counts words in aggregated text files, and analyzes the percentages of positively and negatively valenced words, as well as linguistic and psychological aspects of language. The program matches the LIWC categories with the words in a text file, and computes an overall density score. The word density score for all written texts produced by participants is summarized in Table 1.

**Table 1:** Language Inventory Word Count (LIWC) categories in the present study.

**Categories**	**Abbreviation**	**Example**	**Word density**	**Word density**
		**words**	**(sample)**	**(LIWC2015 norms)**
Words/sentence	WPS	–	22.22 (not %)	17.40 (not %)
Words >6 letters	SIX	–	19.85	15.60
**Linguistic dimensions**				
Personal pronoun	PPron	I, them, her	12.03	9.95
1st singular	I	I, me, mine	10.74	4.92
1st plural	We	We, us, our	0.34	0.72
Adverbs	adverb	Very, really	6.08	5.27
Negations	Negate	No, not, never	1.95	1.66
**Psychological processes**				
Affective processes	Affect	Happy, cried	7.49	5.57
Positive emotions	Posemo	Love, nice	3.95	3.67
Negative emotions	Negemo	Hurt, ugly	3.44	1.84
Anxiety	Anx	Worried, fear	1.90	0.31
Anger	Anger	Hate, kill	0.21	0.54
Sadness	Sad	Crying, grief	0.63	0.41
Social processes	Social	Mate, talk	4.08	9.74
Family	Family	Dad, aunt	0.15	0.44
Friends	Friends	Buddy, neighbor	0.24	0.36
Certainty	Certain	Always, never	2.19	1.35
Past focus	Focuspast	Ago, did, talked	5.72	4.64
Present focus	Focuspres	Today, is, now	9.52	9.96
Future focus	Focusfut	May, will, soon	0.94	1.42
Time	Time	End, until, season	6.37	5.46
Work	Work	Jobs, majors	7.01	2.56
Leisure	Leisure	Cook, chat, movie	0.30	1.35
Home	Home	Kitchen, landlord	0.19	0.55
Money	Money	Audit, cash, owe	0.33	0.68
Religion	Relig	Altar, church	0.05	0.28
Death	Death	Bury, coffin, kill	0.02	0.16
Swear words	Swear	Fuck, damn	0.04	0.21

#### 3.6.2. Correlational Analysis

Bivariate correlations were computed between self-reported GAD, BIS-sensitivity, BAS-sensitivity, and the LIWC categories captured in the written texts produced by the participants.

#### 3.6.3. GAD Prediction

Classification algorithms were applied for GAD recognition considering two feature sets: the LIWC outcomes, and the concatenation of the LIWC outcomes and the BIS/BAS scores per scale item (i.e., feature level fusion). The open source implementation of four commonly used classifiers from the Scikit-learn toolkit (58) was utilized: Support Vector Machine with linear kernel (SVM), Logistic Regression (LR), Naive Bayes (NB), and Random Forest (RF).

Experiments were performed in a 10-fold cross-validation setup, which was repeated 10 times. Each repetition implied splitting the data into new folds, and these folds were preserved for each classification setup to ensure comparable results. This procedure resulted in 100 data points for test performance for each classification pipeline. Averaging across results from multiple train-test splits provides a more robust estimation of the model's performance on unseen data compared to a single split. Within each repetition of the cross-validation, an additional inner 5-fold cross-validation on the training set was performed to facilitate hyperparameter tuning. The best performing estimators were identified through a grid search where the inner cross-validation folding served as development set. To account for the imbalanced data distribution, the balanced accuracy was chosen as performance metric within the grid search. Prediction results are presented in terms of unweighted (macro) average precision (UAP) and recall (UAR).

As a preprocessing step the features were scaled to [0,1]. Two feature selection procedure were explored: univariate feature selections and permutation feature selection. For univariate feature selection, the *K* most informative features were selected using the SelectKBest algorithm from Scikit-learn based on univariate statistical tests (χ^2^), and the remaining features were discarded. The value of k was varied for each classification pipeline and the best *K* for each experiment was chosen. Permutation-based features selection computes the decrease in a model's performance when a single feature value is shuffled randomly (59). This procedure breaks the relationship between a feature and the target, hence performance decreases corresponding to the extent to which the model depends on that feature.

## 4. Results

### 4.1. Correlational Results

Table 2 depicts the correlations between GAD, BIS-sensitivity, BAS-sensitivity, and the LIWC categories identified in the participants' expressive writings. Significant positive correlations were found between GAD and the following LIWC categories: negations, sadness, personal pronouns, present focus, social processes, family, negative emotions, and anger. Significant negative correlations were obtained for GAD and longer words, positive emotions, and focus on the future (Table 2, column 3). The remaining columns present the correlation coefficients between GAD and the BIS, BAS, and LIWC category variables. For participants who scored high on GAD, a significant positive correlation was found between anxiety and BAS. For participants who scored low on GAD, significant negative correlations were observed between BAS, affective processes, and negative emotions. A positive correlation was found between BAS and family. No significant correlations were obtained between LIWC category, BIS and (high or low) GAD (Table 2, columns 4–7).

**Table 2:** Language Inventory Word Count (LIWC) and Behavioral Inhibition System (BIS ) and Behavioral Approach System (BAS) correlations for Generalized Anxiety Disorder (GAD) (*p < 0.05 level, **p < 0.01 level; two-tailed).

	**Abbreviation**	**GAD**	**BIS-sensitivity**		**BAS-sensitivity**	
			**No**		**No**	
			**GAD**	**GAD**	**GAD**	**GAD**
Words/sentence	WPS	−0.03	−0.05	0.04	0.14	−0.04
Words >6 letters	SIX	−0.18^*^	−0.03	−0.09	0.14	0.04
**Linguistic dimensions**						
Personal pronoun	PPron	0.19^*^	0.11	0.18	−0.14	0.27
1st singular	I	0.10	0.08	0.17	−0.08	0.19
1st plural	We	0.08	0.05	−0.03	−0.09	0.12
Adverbs	adverb	−0.06	−0.01	−0.01	0.09	−0.13
Negations	Negate	0.30^**^	0.18	0.02	0.03	−0.07
**Psychological processes**						
Affective processes	Affect	−0.04	0.02	0.12	−0.29^**^	0.16
Positive emotions	Posemo	−0.20^*^	0.05	−0.02	−0.13	0.10
Negative emotions	Negemo	0.18^*^	−0.06	0.17	−0.26^**^	0.12
Anxiety	Anx	−0.12	−0.09	0.12	−0.19	0.28^*^
Anger	Anger	0.26^**^	0.01	0.05	−0.08	0.01
Sadness	Sad	0.30^**^	0.00	0.08	−0.13	−0.16
Social processes	Social	0.23^**^	0.19	0.11	0.01	0.08
Family	Family	0.25^**^	0.08	0.09	0.22^*^	−0.08
Friends	Friends	−0.01	0.17	−0.07	−0.02	.00
Certainty	Certain	−0.08	−0.14	0.05	0.01	0.22
Past focus	Focuspast	−0.09	0.10	−0.16	0.06	0.07
Present focus	Focuspres	0.18^*^	0.01	0.21	−0.13	0.07
Future focus	Focusfut	−0.17^*^	−0.04	−0.07	−0.17	−0.06
Time	Time	−0.14	−0.11	−0.19	0.06	0.01
Work	Work	−0.14	0.11	−0.14	0.09	0.09
Leisure	Leisure	0.04	0.03	0.13	0.16	−0.23
Home	Home	0.09	−0.07	0.13	−0.12	−0.23
Money	Money	0.05	−0.18	−0.01	−0.05	−0.01
Religion	Relig	.10	−0.10	0.13	−0.32	−0.06
Death	Death	0.09	−0.08	−0.06	0.21	−0.10
Swear words	Swear	0.09	0.01	0.06	0.06	0.12

### 4.2. Classification Results

**Figure 1:**
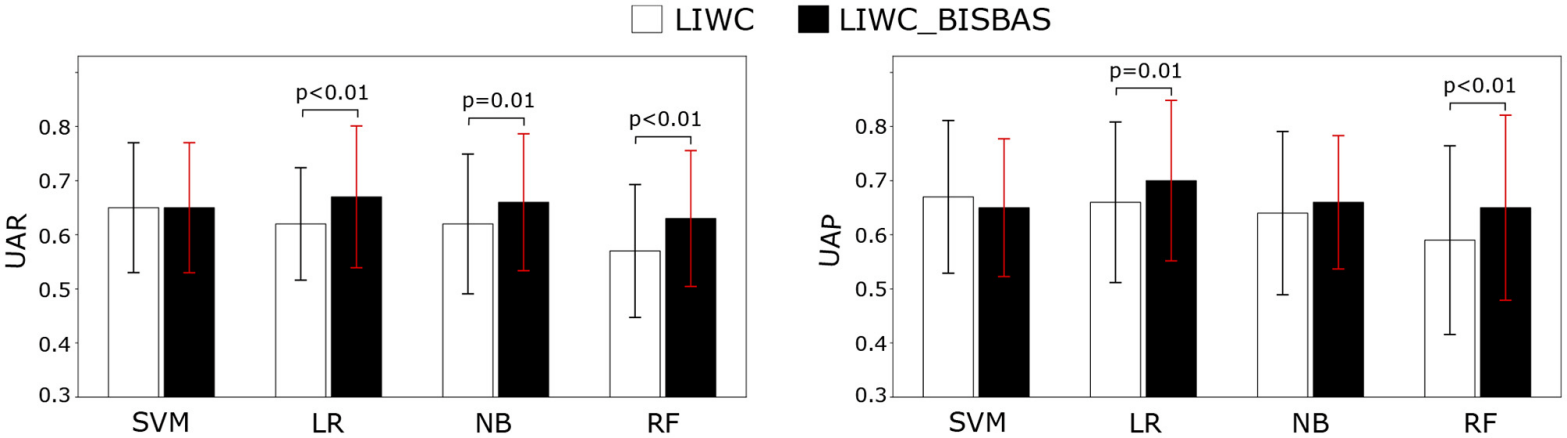
Unweighted average recall (UAR) and unweighted average precision (UAP) for the four classifiers given Language Inventory Word Count (LIWC) features, and for a concatenation of LIWC features, Behavioral Inhibition System and Behavioral Approach System personality features (LIWC_BISBAS), respectively.

Figure 1 presents the unweighted average recall and precision for the best performing classification pipelines tested. The linguistic features as well as their concatenation with the BIS/BAS features predicted GAD well above chance level. The 100 results datapoints from the repeated cross-validation were used as data for Wilcoxon statistical significance test, to compare the performance of the feature sets. The highest performance was obtained when linguistic and BIS/BAS personality features were fused, with one exception for precision of SVM. Furthermore, for all classifiers except for SVM the improvement in recall was statistically significant, while the improvement in precision was significant for LR and NB. This is in line with (60), where increased GAD recognition performance was obtained when facial behavior features were augmented with (Big Five) personality features. Inspection of confusion matrices revealed that prediction of the target class (“anxious”) was always highest in the fused feature set. This is relevant especially from a medical (e-health or m-health) application perspective, when one would not want to miss recognizing the anxious subjects.

The most important features per feature set for all categories based on permutation importance for the RF classifier are outlined in Table 3. For LIWC categories alone (Table 3, column 1), GAD recognition was based primarily on negations, negatively valenced emotion/affect words, and social processes—key ingredients in emotion regulation and behavioral avoidance-based theories on GAD (16). These LIWC categories remained important when BIS/BAS features were included (Table 3, column 2), together with a person's overall BIS and the frequency with which a participant experiences fear or nervousness (BIS scale item 2). This may have been due to the mixed contribution of the behavioral inhibition (BIS) and behavioral approach (BAS) overall scores and individual scale items (Table 3, column 3). The sparse research on GAD and BAS has identified behavioral approach as a potentially important factor in comorbid combinations with GAD, but does not consider it to be a stand-alone factor (36).

**Table 3:** Permutation-based feature importance (Imp) for Generalized Anxiety Disorder (GAD) classification for the Random Forest (RF) model for Language Inventory Word Count (LIWC) categories and for the concatenation of LIWC categories with Behavioral Inhibition System and Behavioral Approach System features (LIWC_BISBAS).

**LIWC**	**Imp**	**LIWC_BISBAS**	**Imp**
L_negate	0.18	BIS Overall	0.18
L_negemo	0.16	L_negate	0.08
L_sad	0.14	L_negemo	0.08
L_social	0.14	L_sad	0.06
L_WPS	0.10	L_focuspast	0.06
L_adverb	0.10	L_work	0.04
L_affect	0.10	BIS (item 2)	0.04
L_posemo	0.10	L_WPS	0.02
L_certain	0.10	L_Sixltr	0.02
L_focusfuture	0.10	L_posemo	0.02

The classifiers in the present study performed better than chance, but they still produced a large amount of error. Analysis of the false positives processed by the classifiers revealed two general conditions under which the classification systems incorrectly identified someone as a person with GAD: First, several people with a self-reported GAD score ≤9 (indicating “no GAD”) had written a text, in which they had recalled their university experience in negative terms (i.e., with negations, negatively valenced emotion words, and under explicit reference to the stress and anxiety that university regulations had elicited in them). Second, a group of “no GAD” people had provided a written contribution on their university experience that was mixed in terms of emotional valence. In those cases, the person had often entered a lengthy and detailed recollection of an anxious and stress-inducing experience, wrapped in between a short positive opener and conclusion on how good the university experience had been after all.

Analysis of the false negatives produced by the classifiers for people with a self-reported GAD score ≥10 (indicating “GAD”) revealed a similar issue. As before, the classification system experienced difficulty in correctly identifying text entries of mixed emotional valence. This was particularly observed in very short written contributions (comprising 2–3 condensed sentences), where a lengthier positive sentence about the good sides of university life often overruled a shorter, negative sentence, in which an anxious university experience had been recollected. For future work in this direction, this seems to suggest that researchers should seek to collect larger portions of written text so as to avoid the occurrence of false negatives in classification.

## 5. Discussion

### 5.1. Strengths and Limitations

The main objective of this study was to investigate if Generalized Anxiety Disorder can be inferred from the language people use in expressive writing. Evidence was generated among a population of undergraduate students that GAD can, indeed, be successfully predicted from an anxiety-focused narrative writing prompt. The second objective of this study was to explore, if accounting for individual differences in inhibition and approach sensitivity increases the accuracy of GAD prediction from written text. For all classifiers used, GAD prediction accuracy was slightly higher when features associated with inhibition, approach and language were combined. In the absence of those features, the results hint toward the possibility that negatively valenced emotion words suffice for GAD recognition from language. This result would make sense from the perspective of emotion-based models of GAD (10, 21), and be consistent with two similar findings on the relation between GAD and language usage (31, 32). When the writer's individual sensitivity in behavioral inhibition and approach were included, a writer's overall BIS-sensitivity score emerged as the most important feature for GAD classification, followed by the negative emotion words. This is theoretically in line with behavioral avoidance models of worry and GAD (5, 7, 8, 11, 26). Inclusion of behavioral inhibition and approach scores thus contribute to the classification of GAD, and could be used in cases, in which it is possible to collect self-report measurements together with textual data.

It may be argued that the specific framing of the writing task in this study—i.e., to recall and describe an anxious moment in university life—primed the student participants toward anxiety in their textual contributions. Instructions to write about an unpleasant (vs. beautiful) moment in life have, indeed, been used in laboratory research with undergraduate students to temporarily induce avoidance-related (vs. approach) states (61–63). It should be emphasized, however, that writing-based priming interventions of approach-avoidance states are highly directive in nature. It does not suffice to simply ask participants to write about an unpleasant or pleasant experience in life. Participants in such studies must be provided with an obligatory list of approach or avoidance-related elements to be incorporated in the narrative. To illustrate: in order to induce a temporary avoidance state, participants are forced to describe in detail their growing awareness of an apparent threat, their subsequent attempts to escape it (63), chronicle how attempts to avoid the threat failed, and discuss the unpleasant consequences of the failure (61, 62). Participants in the present study were not subjected to such a forced-plot-avoidance prime; they received full freedom to recall an anxious experience of their own choice, and to describe it in the way they wanted.

Moreover, even though approach-avoidance motivation and positive-negative affect are theoretically related to each other, they are not identical, conceptually. This is, why the studies that employed the forced writing instructions discussed above all explored the potential overlap between the approach-avoidance manipulations and emotion states (as expressed in participant responses on the items of a positive and negative affect scale) in supplementary analyses. All those studies showed that triggering temporary avoidance states in participants leaves their self-reported negative mood states unaffected. To be precise, writing following the instructions of an explicit avoidance prime had no significant impact on the participant's self-reported negative (“nervous,” “sad,” “disappointed,” “tense,” “depressed”) emotion states. Put differently, even subjecting undergraduate student participants to a bold and explicit narrative writing prime of anxious avoidance does not lead them to automatically also self-report alleviated negative emotion states; all it triggers is the intended motivation state (61, 63). That negative emotion words featured prominently in the writings of a specific sub-group of undergraduate students—after provision of a more subtle writing assignment—may thus not be caused by task instructions biased toward anxious avoidance, but due to linguistic markers associated with GAD.

It would, therefore, be particularly interesting to investigate to what extent the linguistic markers for GAD found in this research are present in the free texts that people produce on other—online and offline—occasions. Narratives as disclosed on social media, online health forums and in personal diaries capture the person's words and expressions in natural habitat, and may produce realistic (ecologically valid) text corpora under unique psychosocial circumstances (48, 51). The written healthcare narratives shared on online mental health forums, where users often are transparent about the mental disorder(s) they are diagnosed with (64–66), would be relevant, but also less topical free texts should be insightful. By definition, people with GAD experience symptoms like restlessness, fatigue, concentration issues, irritability, muscle tension and sleep disturbance very frequently in their daily life (3). Given that GAD is formally defined in terms of extreme and uncontrollable worrying behavior “on multiple everyday events” (2), it stands to reason that linguistic markers for GAD generalize beyond the recall of anxious university experiences, and will also feature prominently—and frequently—in such alternative narratives. However, the free-floating anxiety of people with GAD as expressed in such user-generated free text entries may be heterogeneous on many different (personal, situational, environmental) levels . Inclusion of individual indicators for mental health—such as self-reported personality and individual differences— would be very helpful in capturing some of the heterogeneity under those circumstances (67).

The limitations of this work are the following: First, our results have their origins in a single study with a small sample size, rather than in a larger series of replications. It remains to be seen to what extent our findings generalize to the wider population. For instance, the undergraduate students in the present study were in the final stages of their educational programs. The worries of these students primarily centered on the quality of their performance as a student, their career prospects, and interpersonal conflicts (such as conflicts with thesis supervisors). The nature of their anxious experiences may have differed from those experienced by younger students in earlier stages of education. On the one hand, the narrative method is argued to infer generalizable motives and emotion states from the writings people produce , independent from topic and setting (68). This would suggest that the findings obtained in the present study also apply to younger people in educational environments. Research on worry and GAD in children and adolescents between 5 and 16 years old largely supports this point of view. Alleviated worrying about achievements and interpersonal conflicts in school is also prevalent in young children with GAD (69). On the other hand, adults and children with GAD also differ from each other on a small number of unique focal points. Specifically, children with GAD turn out to worry about the health of other people around them (69). They do not (yet) hold a firm, positive belief about worry as a constructive mechanism for emotion regulation (70). It seems likely that these two aspects of worry render the writings of young children with GAD about experiences in school somewhat different from those of older undergraduate students with GAD.

Second, the extent to which our results are representative to older populations in society is open to debate. GAD has been identified as a common mental disorder among the elderly (71). The considerable distress that anxiety causes in older people has been documented [cf., (72, 73)]. In principle, in case GAD became chronic over time (1), it should remain visible in stable — trait-like — patterns of behavior (cf., 19) also at older age. If this is true, asking older adults to recollect in writing an anxious experience in life should yield similar linguistic results as in the present study. However, recall of an anxious experience in the past may be challenging for some older adults due to memory impairments, and lead to inconsistent and unreliable accounts. Attempts to assess elderly adults on a present anxious experience (felt “right now” rather than experienced in the past) have proven unsatisfactory in as far as anxiety-related states concern (74).

Moreover, older people often find it difficult to understand and adequately respond to assessments in questionnaire format (75). Some argue that older people are less inclined to confess their inner worries and anxieties to anonymous others, are more likely to provide desirable answers, or lack the mental and practical skills to clearly express themselves in self-report questionnaires and diagnostic face-to-face interviews (71). It has, for instance, proven difficult to screen elderly individuals on GAD using established measurement instruments such as the GAD-7. Proposals have even been made to lower the cut point in the GAD-7 from 10 to 5 for older adults to control for their under-reporting of anxious worries (71). Where modification of the temporal focus in writing instructions would complicate comparisons of language usage between older and younger adults with GAD, drastic psychometric adjustment in the cut point for identification of GAD for the elderly—but not for the younger population—would render comparisons impossible. Attempts to replicate the present findings in older populations may, therefore, be a challenging undertaking.

### 5.2. Future Research and Conclusions

Our work should be perceived as a first step toward linguistics-based anxiety recognition. Given the modest sample size of the present study, future work should seek to expand data collection so as to create more stable prediction results that are generalizable to other—younger and older—populations of people with GAD within and beyond the educational setting. It is important to do so, because the quest for unique behavioral markers of GAD is far from a resolved issue. In fact, the search for verbal linguistic markers that exclusively apply to GAD has only just begun.

The search for unique (behavioral and verbal linguistic) markers of GAD is a complicated endeavor due to the documented comorbidity of GAD with several other mental disorders. First and foremost, GAD is often comorbid with major depression [cf., (11, 16, 53, 76, 77)]. Unlike other anxiety disorders, an alleviated presence of self-focused attention and negative affect characterize both GAD and major depression (22, 30). The overlap in symptoms between the two mental disorders is such that GAD and major depression have been argued to be two sides of the same coin, different only in stage of development [cf., (78, 79)]. Second, GAD often is comorbid with social anxiety disorder (SAD) (22, 77). These two anxiety disorders are highly overlapping in as far as maladaptive, avoidance-based, emotion regulation strategies in response to distress concern [cf., (80, 81)]. Even the worrying behavior so prevalent in GAD is also found in this other anxiety disorder—albeit at lower intensity and lower frequency [cf., (82)]. It has been argued that the difference of GAD (vs. SAD) lies in the combination of emotion intensity and maladaptive strategies for emotion regulation (23). Future work should, therefore, seek to not only identify verbal-linguistic markers that uniquely apply to GAD, but also look into the intensity of words expressing negative emotions and social process.

Another interesting issue to address is the trustworthiness of verbal descriptions. Prior work shows that written diary entries from people with GAD contain inaccurate descriptions of experienced emotions, due to episodes of emotion dysregulation (16). This may have repercussions on the diagnostic value of text-based health applications for anxiety recognition. From an affective computing point of view, such applications could be complemented with functionalities for multi-modal behavior analysis and physiological response measurement. This would compensate for the fact that anxiety, in general, is inferred not only from verbal descriptions, but also from behavioral and physiological responses (83).

In the longer term, the insights of this and future studies into linguistics-based GAD recognition could lead to the development and implementation of new digital health technologies to remotely screen for GAD. In clinical setting, participation and engagement are key determinants in determining the success or failure of digital health solutions. Mair et al. (84) emphasize that the potential benefits of a new e-health application should have to make sense to all users involved (healthcare professionals, healthcare organizations, and service users), and that all stakeholders involved must be willing to actively engage with—and critically assess—the new technology. Implementation plans should include repeated assessment of the ease of use and willingness to accept the technology in clinical practice (85) as well as evaluation of the perceived technology readiness level. This “propensity to embrace and use [the technology] for accomplishing goals at home, life, and at work” [(86), p. 308] should also include thorough assessment of ethical and legal issues that may arise from using the e-health application in clinical setting (87). This requires execution of pilot projects in iteration so as to collect and account for the individual preferences of each group of stakeholders involved (cf., 88). The iterative process should go on until all groups agree on the desirability to implement the application into the larger healthcare system (89). In order to successfully implement new e-health solutions within the larger healthcare system, such studies must be grounded in state-of-the-art scientific knowledge (88). We hasten to emphasize that it is of vital importance to first resolve conceptual issues regarding identification of unique linguistic markers of GAD and methodological issues involving generalizability to other populations before this iterative process of turning knowledge to action (90) is set in motion.

In conclusion, the present study set a first step toward linguistics-based anxiety recognition. It is our hope that the results of this paper will eventually contribute to remote identification of generalized anxiety disorder—persistent anxious worry in the absence of unique behavioral markers—from expressive writings.

## Data Availability Statement

The raw data supporting the conclusions of this article will be made available by the authors, without undue reservation.

## Ethics Statement

The studies involving human participants were reviewed and approved by Human Research Ethics Committee, Delft University of Technology. The patients/participants provided their written informed consent to participate in this study.

## Author Contributions

LR, MM, and FB developed the study. MM collected the data and conducted the expert interviews. LR and MM conducted the data analyses. IL ran the prediction models and reported the classification results. LR had the lead in reviewing the literature, writing the paper, and drafting different versions of the paper that were reviewed and (substantially) edited by MM, IL, and FB. LR, MM, IL, and FB approved the final version. All authors contributed to the article and approved the submitted version.

## Conflict of Interest

The authors declare that the research was conducted in the absence of any commercial or financial relationships that could be construed as a potential conflict of interest.

## Publisher's Note

All claims expressed in this article are solely those of the authors and do not necessarily represent those of their affiliated organizations, or those of the publisher, the editors and the reviewers. Any product that may be evaluated in this article, or claim that may be made by its manufacturer, is not guaranteed or endorsed by the publisher.
